# Impact of initial treatment and prognostic factors on postprogression survival in *BRAF*-mutated metastatic melanoma treated with dacarbazine or vemurafenib ± cobimetinib: a pooled analysis of four clinical trials

**DOI:** 10.1186/s12967-020-02458-x

**Published:** 2020-08-03

**Authors:** Paolo A. Ascierto, Antoni Ribas, James Larkin, Grant A. McArthur, Karl D. Lewis, Axel Hauschild, Keith T. Flaherty, Edward McKenna, Qian Zhu, Yong Mun, Brigitte Dréno

**Affiliations:** 1 Istituto Nazionale Tumori IRCCS Fondazione G. Pascale, Via Mariano Semmola, 80131 Naples, Italy; 2 Jonsson Comprehensive Cancer Center, University of California at Los Angeles, 100 Medical Plaza Driveway #550, Los Angeles, CA 90095 USA; 3 The Royal Marsden NHS Foundation Trust, 203 Fulham Road, Chelsea, London, SW3 6JJ UK; 4 Peter MacCallum Cancer Centre, 305 Grattan Street, East Melbourne, VIC 3000 Australia; 5 University of Melbourne, Parkville, VIC Australia; 6 University of Colorado Comprehensive Cancer Center, 1665 Aurora Court, Aurora, CO 80045 USA; 7 University Hospital Schleswig-Holstein, Rosalind-Franklin-Str. 7, 24105 Kiel, Germany; 8 Massachusetts General Hospital, 55 Fruit Street, Yawkey 9E, Boston, MA 02114 USA; 9 Genentech, Inc., 1 DNA Way, South San Francisco, CA 94080 USA; 10 Nantes University, 1 Place Alexis Ricordeau, 44093 Nantes Cedex 1, France

**Keywords:** Vemurafenib, Cobimetinib, Dacarbazine, Melanoma, Survival analysis

## Abstract

**Background:**

We sought to identify patient subgroups with distinct postprogression overall survival (ppOS) outcomes and investigate the impact of original treatment assignment and initial postprogression treatment (ppRx) on ppOS.

**Methods:**

Recursive partitioning analysis (RPA) was performed to model relationships between prespecified covariates and ppOS in patients with *BRAF*^V600^-mutated metastatic melanoma who had experienced progressive disease (PD) following treatment with cobimetinib plus vemurafenib, vemurafenib monotherapy, or dacarbazine in the BRIM-2, BRIM-3, BRIM-7, and coBRIM studies. Prognostic subgroups identified by RPA were then applied to pooled treatment cohorts. The primary endpoint was ppOS, defined as time from first PD to death from any cause.

**Results:**

RPA identified baseline lactate dehydrogenase (LDH), baseline disease stage, Eastern Cooperative Oncology Group performance status at PD, and ppRx as significant prognostic factors for ppOS. Median ppOS was longest in patients with normal baseline LDH, stage M1c disease at baseline, and ppRx with immunotherapy or targeted therapy (12.2 months; 95% CI 10.3–16.1) and shortest in those with elevated baseline LDH > 2 × upper limit of normal (2.3 months; 95% CI 1.8–2.7). Original treatment assignment did not impact ppOS. Across treatment cohorts, patients treated with immunotherapy or targeted therapy after PD had better ppOS than those given other treatments.

**Conclusion:**

A combination of factors at baseline (LDH, disease stage) and PD (performance status, ppRx) impact ppOS outcomes. ppRx with immunotherapy or targeted therapy is an independent prognostic factor for improved overall survival following progression regardless of original treatment.

*Trial registration* The trials included in this analysis are registered with ClinicalTrials.gov: NCT00949702 (BRIM-2), NCT01006980 (BRIM-3), NCT01271803 (BRIM-7), and NCT01689519 (coBRIM).

## Background

Vemurafenib monotherapy and cobimetinib plus vemurafenib have improved survival in patients with *BRAF*^V600^-mutated metastatic melanoma [1–7]. Extended follow-up of clinical studies evaluating BRAF inhibitor (BRAFi) monotherapy or combined BRAFi and MEK inhibitor (MEKi) shows a plateau in overall survival (OS) curves after approximately 3 years [8–11]. A similar plateau in OS is observed with ipilimumab [12]. These observations suggest that a subgroup of patients with metastatic melanoma have good long-term survival prognosis. Prognostic factors for survival in patients with metastatic melanoma include disease stage (number/location of metastases), baseline lactate dehydrogenase (LDH), and baseline Eastern Cooperative Oncology Group performance status (ECOG PS) [13, 14]. Recent analyses suggest that LDH remains the most important prognostic factor for survival in patients treated with BRAFi and/or MEKi or immunotherapy, with particularly poor outcomes being observed in patients with LDH elevated > 2 × upper limit of normal (ULN) [15–18]. However, the impact on survival outcomes of clinical and disease-related variables and treatment following progression are poorly understood. Some reports suggest that prior BRAFi treatment is associated with inferior response to subsequent immunotherapy [19–23], whereas others suggest that the efficacy of immunotherapy is unaffected by previous BRAFi therapy [24–27]. Insight into the impact of clinical characteristics and subsequent treatment on survival following progressive disease (PD) may inform treatment decisions in the management of patients with metastatic melanoma.

The objectives of this analysis were to 1) identify patient subgroups with distinct postprogression overall survival (ppOS) outcomes and 2) investigate the impact of original assigned treatment (cobimetinib plus vemurafenib, vemurafenib monotherapy, or dacarbazine) and initial postprogression treatment (ppRx) on ppOS in patients with *BRAF*^V600^-mutated metastatic melanoma.

## Methods

### Study design and participants

Data were pooled from the BRIM-2 (NCT00949702) [1], BRIM-3 (NCT01006980) [3, 4], BRIM-7 (NCT01271803) [5], and coBRIM (NCT01689519) [6, 7] studies. Detailed methods have been previously reported. Briefly, BRIM-2 was an open-label, multicenter phase 2 trial of oral vemurafenib 960 mg twice daily [2]. BRIM-3 was an open-label, multicenter, randomized phase 3 trial of oral vemurafenib 960 mg twice daily compared with intravenous dacarbazine 1000 mg/m^2^ every 3 weeks [3, 4]. BRIM-7 was an open-label, multicenter phase 1b dose-escalation study of oral cobimetinib 60, 80, or 100 mg once daily on a 14 days on/14 days off schedule (14/14), a 21 days on/7 days off schedule (21/7), or continuously (28/0) combined with oral vemurafenib 720 or 960 mg twice daily [5]. coBRIM was a randomized, double-blind phase 3 study of the combination of oral cobimetinib 60 mg once daily on a 21/7 schedule combined with oral vemurafenib 960 mg twice daily compared with placebo plus vemurafenib [6, 7]. Key eligibility criteria were similar across trials; eligible patients were aged ≥ 18 years with unresectable stage IIIC or IV melanoma harboring a *BRAF*^V600^ mutation, ECOG PS of 0–1, and adequate organ function. BRIM-3 and coBRIM enrolled previously untreated patients only, whereas BRIM-2 enrolled patients who had received prior systemic treatment for advanced disease, and BRIM-7 enrolled both previously treated and untreated patients. All four trials allowed enrollment of patients with previously treated, stable brain metastases.

All eligible patients (defined by each study protocol) who had PD at the time of data cutoff were included in analyses regardless of treatment assignment. Patients with missing data for covariates of interest were excluded. For BRIM-7, only BRAFi-naive patients were included in the analysis.

### Statistical analysis

#### Recursive partitioning analysis

Recursive partitioning analysis (RPA) is a statistical method that creates a decision tree by classifying patients into subgroups, defined by independent prognostic factors, with statistically distinct survival probabilities. RPA was undertaken to model relationships between prespecified covariates and ppOS in patients who had experienced PD at the time of data cutoff (BRIM-2: February 1, 2012; BRIM-3: July 8, 2015; BRIM-7: December 2, 2015; coBRIM: August 28, 2015).

Covariates were prespecified in study protocols and/or identified as potential prognostic factors from peer-reviewed medical literature. Prespecified baseline covariates were age (< 65 vs ≥ 65 years), sex (male vs female), race (white vs nonwhite), geographic region (North America, Europe, or Australia/New Zealand/other), ECOG PS (0, 1, or 2), disease stage (unresectable IIIC, M1a, M1b, M1c), LDH (normal, elevated ≤ 2 × ULN or elevated > 2 × ULN), sum of longest diameters (SLD) of target lesions, histologic subtype (acral lentiginous, lentigo maligna, nodular, superficial spreading, or other), prior adjuvant therapy (yes vs no), and original treatment assignment (cobimetinib plus vemurafenib, vemurafenib monotherapy, or dacarbazine). Prespecified covariates at the time of PD were best response before PD, LDH (normal, elevated ≤ 2 × ULN, or elevated > 2 × ULN), ECOG PS (0, 1, or 2), metastasis (new lesion vs no new lesion; central nervous system [CNS] vs non-CNS), SLD, enlargement of baseline lesions, maximum change in SLD, time from maximum change in SLD to PD, absolute lymphocyte count (normal, low, or elevated), ratio of absolute neutrophil count to absolute lymphocyte count, and initial ppRx (immunotherapy/targeted therapy vs other).

Initial ppRx was defined as ≥ 1 dose of ipilimumab, pembrolizumab, nivolumab, or similar therapy within 90 days after PD (immunotherapy); ≥ 1 dose of vemurafenib, cobimetinib, dabrafenib, trametinib, or similar therapy within 90 days after PD (targeted therapy); or receipt of any therapy other than immunotherapy or targeted therapy, both immunotherapy and targeted therapy, or no treatment within 90 days after PD (other).

RPAs used a unified framework for conditional inference (permutation tests) using censored response variables to avoid bias in selection of covariates and minimize overfitting of the data [28]. This approach ensures a right-sized tree with no need for pruning or cross-validation. The global null hypothesis of independence between any of the prespecified input variables and survival was tested. If the null hypothesis could not be rejected, the analysis was stopped; otherwise, the covariate most strongly associated with survival was identified based on univariate *P* values and a binary split was implemented. These steps were repeated until no further covariates with a significant association with survival could be distinguished. The stop criterion was based on either multiplicity-adjusted univariate *P* values or a prespecified threshold value of the test statistic (both criteria were maximized using 1—*P* value).

#### ppOS

The primary endpoint for this analysis was ppOS, defined as time from first PD to death from any cause, estimated using the Kaplan–Meier method. ppOS was evaluated using updated data in patients who had experienced PD at the time of updated data cutoff (BRIM-2: February 1, 2012; BRIM-3: July 8, 2015; BRIM-7: July 10, 2017; coBRIM: October 13, 2017). Prognostic subgroups identified by RPA were applied to the total pooled population and to pooled treatment cohorts (cobimetinib plus vemurafenib, vemurafenib monotherapy, and dacarbazine). Patients who did not die were censored at data cutoff.

## Results

### RPA

At the time of data cutoff for the RPA, 1004 patients had experienced PD; after excluding patients with missing data, 809 patients were included in the RPA. Demographic and clinical characteristics were comparable across the pooled population and pooled treatment cohorts (Table 1).

**Table 1:** Demographic and clinical characteristics for patients included in the RPA

Characteristic	All pooled patients N = 1004	Cobimetinib + vemurafenib n = 184	Vemurafenib monotherapy n = 555	Dacarbazine n = 265
Age, n (%)	(n = 1004)	(n = 184)	(n = 555)	(n = 265)
< 65 y	758 (75.5)	142 (77.2)	405 (73.0)	211 (79.6)
≥ 65 y	246 (24.5)	42 (22.8)	150 (27.0)	54 (20.4)
Sex, n (%)	(n = 1004)	(n = 184)	(n = 555)	(n = 265)
Male	600 (59.8)	117 (63.6)	335 (60.4)	148 (55.8)
Female	404 (40.2)	67 (36.4)	220 (39.6)	117 (44.2)
Race, n (%)	(n = 1004)	(n = 184)	(n = 555)	(n = 265)
White	971 (96.7)	167 (90.8)	539 (97.1)	265 (100.0)
Nonwhite	33 (3.3)	17 (9.2)	16 (2.9)	0
Region, n (%)	(n = 1004)	(n = 184)	(n = 555)	(n = 265)
North America	270 (26.9)	47 (25.5)	163 (29.4)	60 (22.6)
Europe	590 (58.8)	108 (58.7)	314 (56.6)	168 (63.4)
Australia/New Zealand/Others	144 (14.3)	29 (15.8)	78 (14.1)	37 (14.0)
Baseline disease stage, n (%)	(n = 1004)	(n = 184)	(n = 555)	(n = 265)
Unresectable IIIC, M1a, or M1b	332 (33.1)	57 (31.0)	184 (33.2)	91 (34.3)
M1c	672 (66.9)	127 (69.0)	371 (66.8)	174 (65.7)
Baseline ECOG PS, n (%)	(n = 1001)	(n = 182)	(n = 554)	(n = 265)
0	651 (65.0)	129 (70.9)	343 (61.9)	179 (67.5)
1	350 (35.0)	53 (29.1)	211 (38.1)	86 (32.5)
Baseline LDH, n (%)	(n = 942)	(n = 173)	(n = 523)	(n = 246)
Normal	495 (52.5)	82 (47.4)	276 (52.8)	137 (55.7)
Elevated ≤ 2 × ULN	290 (30.8)	61 (35.3)	154 (29.4)	75 (30.5)
Elevated > 2 × ULN	157 (16.7)	30 (17.3)	93 (17.8)	34 (13.8)
Baseline liver metastasis, n (%)	(n = 1001)	(n = 184)	(n = 552)	(n = 265)
Yes	375 (35.7)	69 (37.5)	199 (36.1)	89 (33.6)
No	644 (64.3)	115 (62.5)	353 (63.9)	176 (66.4)
ECOG PS at PD, n (%)	(n = 926)	(n = 166)	(n = 519)	(n = 241)
0	489 (52.8)	108 (65.1)	266 (51.3)	115 (47.7)
1	356 (38.4)	50 (30.1)	209 (40.3)	97 (40.2)
2	55 (5.9)	4 (2.4)	29 (5.6)	22 (9.1)
3	22 (2.4)	4 (2.4)	13 (2.5)	5 (2.1)
4	4 (0.4)	0	2 (0.4)	2 (0.8)
Postprogression treatment, n (%)	(n = 1004)	(n = 184)	(n = 555)	(n = 265)
Immunotherapy	202 (20.1)	39 (21.2)	114 (20.5)	49 (18.5)
Targeted therapy	58 (5.8)	14 (7.6)	27 (4.9)	17 (6.4)
Other	744 (74.1)	131 (71.2)	414 (74.6)	199 (75.1)
Baseline SLD, mm	(n = 995)	(n = 184)	(n = 546)	(n = 265)
Mean (SD)	87.1 (75.2)	86.2 (63.7)	91.2 (85.6)	79.3 (57.2)
Median (range)	70.0 (9–1310)	72.0 (10–398)	71.5 (9–1310)	66.0 (9–295)

*ECOG PS* Eastern Cooperative Oncology Group performance status, *LDH* lactate dehydrogenase, *PD* progressive disease, *ppOS* postprogression overall survival, *RPA* recursive partitioning analysis, *SLD* sum of longest diameters, *ULN* upper limit of normal

Median postprogression follow-up durations were 5.4 months (interquartile range [IQR], 2.2–12.5 months) for all patients, 4.8 months (IQR, 1.9–10.7 months) for the cobimetinib plus vemurafenib cohort, 5.2 months (IQR, 2.2–12.0 months) for the vemurafenib monotherapy cohort, and 6.5 months (IQR, 2.6–15.6 months) for the dacarbazine cohort.

**Fig. 1:**
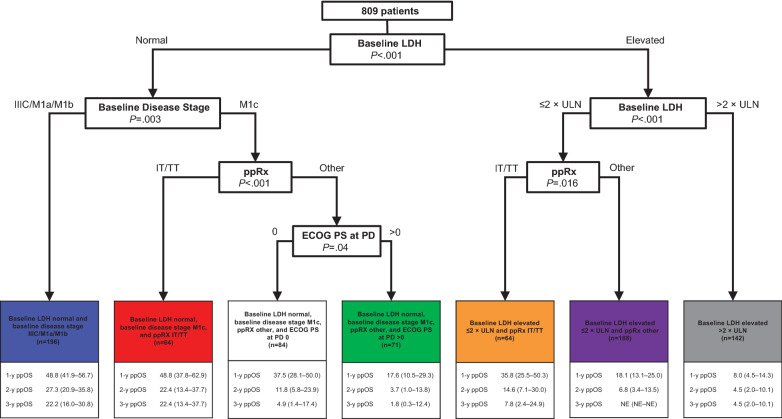
Recursive partitioning analysis for prognostic subgroups in all pooled patients. Recursive partitioning decision tree and ppOS outcomes by identified prognostic subgroups for all pooled patients. Data are presented as percentage (95% CI). *ECOG PS* Eastern Cooperative Oncology Group performance status, *IT* immunotherapy, *LDH* lactate dehydrogenase, *PD* progressive disease, *ppOS* postprogression overall survival, *ppRx* postprogression treatment, *TT* targeted therapy, *ULN* upper limit of normal

In all pooled patients, the RPA identified baseline LDH, baseline disease stage, ECOG PS at PD, and initial ppRx as significant prognostic factors for ppOS, producing seven subgroups with distinct outcomes (Fig. 1). Notably, CNS metastasis at PD was not identified as a significant prognostic factor for ppOS, although this subgroup was relatively small (n = 174). Baseline LDH level was the strongest determinant of ppOS. Baseline disease stage (IIIC/M1a/M1b vs M1c) was identified as a significant prognostic factor among patients with normal baseline LDH but not among those with elevated LDH. Initial ppRx (immune/targeted therapy vs other) was a significant prognostic factor among patients with normal baseline LDH and baseline disease stage M1c, and among those with baseline LDH elevations ≤ 2 × ULN. ECOG PS at the time of PD was prognostic for ppOS among patients with normal baseline LDH, baseline disease stage M1c, and initial ppRx other than immune/targeted therapy. After adjusting for other covariates, including original treatment assignment, initial ppRx with immunotherapy or targeted therapy was associated with longer ppOS than other ppRx. Original treatment assignment (cobimetinib plus vemurafenib, vemurafenib monotherapy, or dacarbazine) was not identified as a prognostic factor for ppOS.

### ppOS

At the time of updated data cutoff for ppOS, 1027 patients had experienced PD; after excluding patients with missing data, 955 patients were included in analyses of ppOS based on RPA-defined prognostic subgroups. Demographic and clinical characteristics remained comparable across cohorts (Additional file 1: Table S1).

Median postprogression follow-up durations were 5.5 months (IQR, 2.2–14.0 months) for all patients, 5.2 months (IQR, 2.1–14.0 months) for the cobimetinib plus vemurafenib cohort, 5.3 months (IQR, 2.2–13.2 months) for the vemurafenib monotherapy cohort, and 6.5 months (IQR, 2.6–15.6 months) for the dacarbazine cohort.

**Fig. 2:**
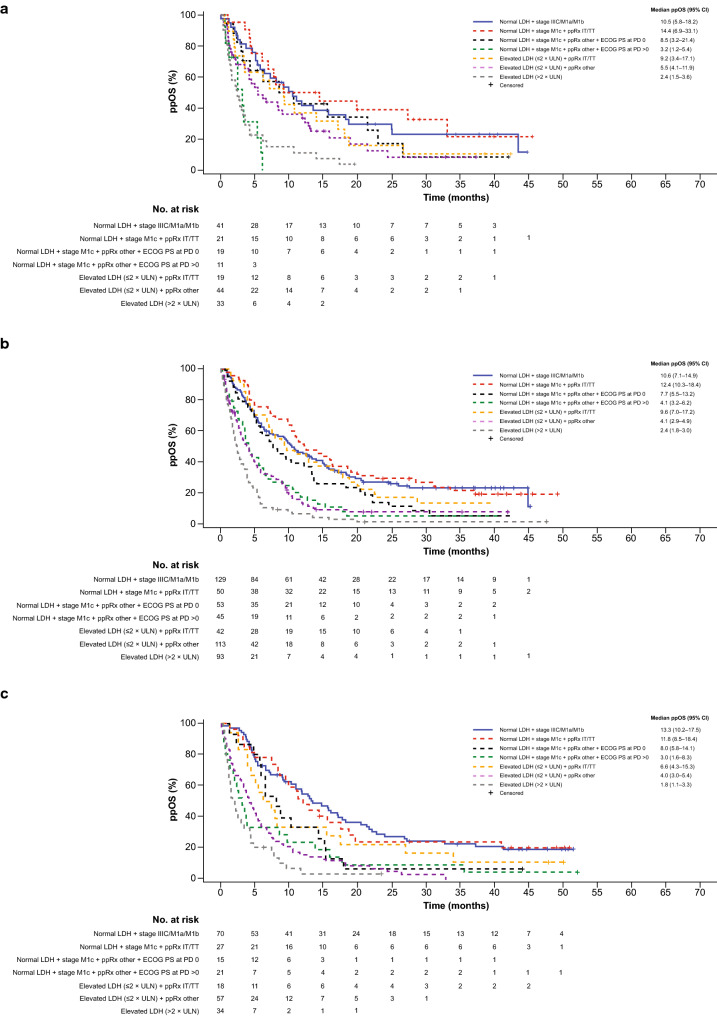
Postprogression overall survival by treatment cohort. Kaplan–Meier curves of ppOS in **a** cobimetinib plus vemurafenib cohort, **b** vemurafenib monotherapy cohort, and **c** dacarbazine cohort. *CI* confidence interval, *ECOG PS* Eastern Cooperative Oncology Group performance status, *IT* immunotherapy, *LDH* lactate dehydrogenase, *NE* not estimable, *PD* progressive disease, *ppOS* postprogression overall survival, *ppRx* initial postprogression treatment, *TT* targeted therapy, *ULN* upper limit of normal

Among all patients, median ppOS was longest in the subgroup with normal baseline LDH, baseline disease stage M1c, and initial ppRx with immunotherapy or targeted therapy (12.2 months; 95% CI 10.3–16.1) and shortest in those with elevated baseline LDH > 2 × ULN (2.3 months; 95% CI 1.8–2.7). Application of prognostic factors identified in the RPA across treatment cohorts (cobimetinib plus vemurafenib, vemurafenib monotherapy, and dacarbazine) revealed similar trends in ppOS (Fig. 2).

ppOS outcomes, stratified by prognostic subgroups identified in the RPA, appeared to be similar across treatment cohorts (Table 2), consistent with the initial RPA demonstrating no impact of original treatment assignment on ppOS. Among patients with normal baseline LDH, baseline disease stage M1c, and initial ppRx with immunotherapy or targeted therapy (the most favorable subgroup), 3-year ppOS rates were 21.7% (95% CI 0.0–44.2) in the cobimetinib plus vemurafenib cohort, 22.3% (95% CI 10.3–34.3) in the vemurafenib monotherapy cohort, and 24.4% (95% CI 7.8–41.1) in the dacarbazine cohort. Among patients with baseline LDH elevations > 2 × ULN (the least favorable subgroup), 3-year ppOS rates were not estimable (NE; 95% CI NE–NE) in the cobimetinib plus vemurafenib cohort, 2.5% (95% CI 0.0–5.8) in the vemurafenib monotherapy cohort, and NE (95% CI NE–NE) in the dacarbazine cohort.

**Table 2:** Postprogression overall survival among all pooled patients and by treatment cohort according to prognostic subgroup

Prognostic subgroup	All patients			Cobimetinib + vemurafenib			Vemurafenib monotherapy			Dacarbazine		
	N (events)	ppOS, median, mo (95% CI)	3-year ppOS, % (95% CI)	N (events)	ppOS, median, mo (95% CI),	3-year ppOS, % (95% CI)	N (events)	ppOS, median, mo (95% CI)	3-year ppOS, % (95% CI)	N (events)	ppOS, median, mo (95% CI)	3-year ppOS, % (95% CI)
Normal LDH + stage IIIC/M1a/M1b	240 (170)	11.2 (9.7–14.0)	23.1 (17.3–28.9)	41 (28)	10.5 (5.8–18.2)	23.1 (8.8–37.5)	129 (88)	10.6 (7.1–14.9)	23.8 (15.5–32.1)	70 (54)	13.3 (10.2–17.5)	22.8 (12.6–32.9)
Normal LDH + stage M1c + ppRx IT/TT	98 (74)	12.2 (10.3–16.1)	23.4 (14.5–32.3)	21 (14)	14.4 (6.9–33.1)	21.7 (0.0–44.2)	50 (39)	12.4 (10.3–18.4)	22.3 (10.3–34.3)	27 (21)	11.8 (8.5–18.4)	24.4 (7.8–41.1)
Normal LDH + stage M1c + ppRx other + ECOG PS at PD 0	87 (72)	8.2 (6.0–10.6)	6.8 (0.7–13.0)	19 (13)	8.5 (3.2–21.4)	8.6 (0.0–24.4)	53 (45)	7.7 (5.5–13.2)	6.0 (0.0–13.6)	15 (14)	8.1 (5.8–14.1)	6.7 (0.0–19.3)
Normal LDH + stage M1c + ppRx other + ECOG PS at PD > 0	77 (71)	3.6 (2.6–4.6)	4.5 (0.0–9.4)	11 (10)	3.2 (1.3–5.4)	0.0 (0.0–0.0)	45 (41)	4.1 (3.2–6.2)	5.7 (0.0–13.0)	21 (20)	3.0 (1.6–8.3)	4.8 (0.0–13.9)
Elevated LDH (≤ 2 × ULN) + ppRx IT/TT	79 (67)	8.1 (6.7–12.9)	11.4 (3.7–19.2)	19 (17)	9.2 (3.4–17.1)	10.5 (0.0–24.3)	42 (34)	9.6 (7.0–17.2)	14.1 (2.8–25.5)	18 (16)	6.6 (4.3–15.3)	11.1 (0.0–25.6)
Elevated LDH (≤ 2 × ULN) + ppRx other	214 (186)	4.2 (3.5–5.0)	5.0 (1.1–8.8)	44 (35)	5.5 (4.1–11.9)	8.4 (0.0–18.9)	113 (95)	4.1 (2.9–4.9)	8.6 (2.8–14.3)	57 (56)	4.0 (3.0–5.4)	0.0 (0.0–0.0)
Elevated LDH (> 2 × ULN)	160 (150)	2.3 (1.8–2.7)	2.7 (0.0–5.5)	33 (29)	2.4 (1.5–3.6)	NE (NE–NE)	93 (89)	2.4 (1.8–3.0)	2.5 (0.0–5.8)	34 (32)	1.8 (1.2–3.3)	NE (NE–NE)

*CI* confidence interval, *ECOG PS* Eastern Cooperative Oncology Group performance status, *IT* immunotherapy, *LDH* lactate dehydrogenase, *NE* not estimable, *PD* progressive disease, *ppOS* postprogression overall survival, *ppRx* postprogression treatment, *TT* targeted therapy, *ULN* upper limit of normal

Across treatment cohorts, patients who received immunotherapy or targeted therapy as initial treatment after progression had better survival outcomes than those given other treatments, even among those with LDH elevations.

### Impact of Initial ppRx on ppOS

**Fig. 3:**
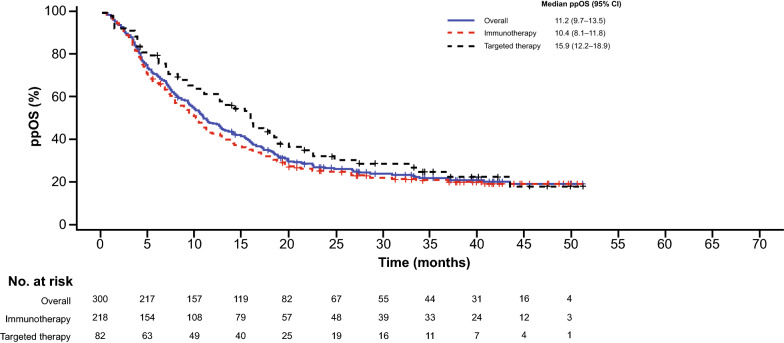
Postprogression overall survival by initial postprogression treatment. Kaplan–Meier curves of ppOS in all pooled patients receiving initial postprogression treatment with immunotherapy or targeted therapy (n = 300). *ppOS* postprogression overall survival

Among 1027 patients with PD at the time of data cutoff, 218 received initial ppRx with immunotherapy and 82 received targeted therapy (Additional file 1: Table S2). Median ppOS was 10.4 months (95% CI 8.1–11.8) in patients who received initial ppRx with immunotherapy and 15.9 months (95% CI 12.2–18.9) in those who received targeted therapy (Fig. 3). ppOS rates at 3 years were 21.1% (95% CI 15.5–26.7) and 25.4% (95% CI 15.0–35.9), respectively.

### ppRx patterns

**Fig. 4:**
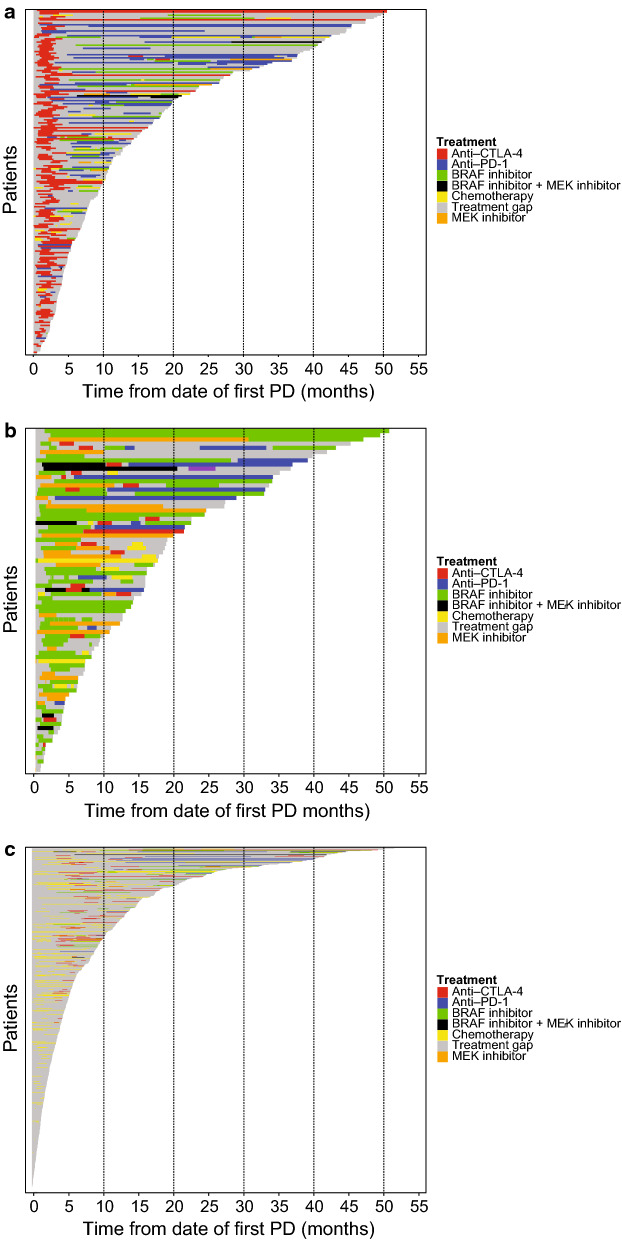
Postprogression treatment patterns by initial postprogression treatment. Postprogression treatment patterns in patients who received initial postprogression treatment with **a** immunotherapy (n = 218), **b** targeted therapy (n = 82), and **c** other (n = 727). *CTLA-4* cytotoxic T lymphocyte–associated antigen 4, *PD-1* programmed death receptor 1

Among patients who received initial ppRx with immunotherapy, initial treatment was ipilimumab in most patients (200 of 218 patients; 91.7%) (Additional file 1: Table S2); subsequent treatment frequently comprised anti–PD-1 therapy or BRAF and/or MEK inhibition (Fig. 4; Additional file 1: Table S3). Among patients who received initial ppRx with targeted therapy, initial treatment was most commonly single-agent BRAFi (61 of 82 patients [74.4%]; Additional file 1: Table S2); targeted therapy was frequently followed by anti–PD-1 or anti–cytotoxic T-lymphocyte–associated protein 4 (CTLA-4) therapy (Fig. 4; Additional file 1: Table S3). Among patients who received other initial ppRx, 220 received chemotherapy and 507 received no treatment (Additional file 1: Table S2). Among the 220 patients who received initial ppRx with chemotherapy, subsequent treatment was most commonly chemotherapy or anti–CTLA-4 therapy (Fig. 4; Additional file 1: Table S3).

Among patients with evidence of additional treatment, median time to next treatment (time from start of initial ppRx to start of next line of ppRx or death) was 4.4 months (95% CI 4.0–4.9) in patients who received initial ppRx with immunotherapy, 6.0 months (95% CI 4.3–7.6) in those who received targeted therapy, and 3.8 months (95% CI 3.3–4.3) in those who received chemotherapy.

## Discussion

In this exploratory analysis of data pooled from four key trials of vemurafenib or cobimetinib plus vemurafenib, a combination of baseline LDH and disease stage, initial ppRx, and ECOG PS at PD were identified as key prognostic factors for ppOS in patients with metastatic melanoma. Original treatment regimen (cobimetinib plus vemurafenib, vemurafenib monotherapy, or dacarbazine) did not appear to impact survival outcomes associated with ppRx.

Previous analyses in patients with metastatic melanoma treated with BRAFi and/or MEKi have consistently identified baseline LDH, ECOG performance status, and extent of disease as prognostic factors for OS [15–18]. In the current analysis, baseline LDH remained the most important prognostic factor for OS following progression. Among patients with normal baseline LDH, baseline disease stage and ECOG PS at the time of PD were also identified as key prognostic factors for ppOS. Additionally, initial ppRx was identified as a statistically significant independent prognostic factor for ppOS on both sides of the regression tree (among patients with normal baseline LDH and those with LDH elevations ≤ 2 × ULN). Interestingly, among patients with normal baseline LDH, patients with stage M1c disease who received initial ppRx with immunotherapy or targeted therapy were able to achieve ppOS that was at least as good as that obtained in patients with stage IIIC/M1a/M1b disease.

Considerable debate exists regarding the optimal sequence of treatment with targeted therapies and immunotherapies for patients with *BRAF*-mutated metastatic melanoma. To date, only low-quality evidence is available to inform treatment sequencing decisions, but randomized studies are currently underway. Retrospective analyses, mostly involving ipilimumab, have suggested that prior treatment with a BRAFi is associated with an inferior response to subsequent immunotherapy [19–21], whereas recent analyses involving nivolumab and pembrolizumab are more controversial [22, 23, 25–27]. In the current analysis, patients who received initial ppRx with immunotherapy or targeted therapy had similar OS outcomes across the cobimetinib plus vemurafenib, vemurafenib monotherapy, and dacarbazine cohorts. After adjusting for other covariates, ppRx with immunotherapy or targeted therapy was associated with longer ppOS than ppRx with other treatments regardless of original treatment assignment. Furthermore, observed ppOS outcomes in patients receiving initial ppRx with immunotherapy in this study (92% ipilimumab) appeared to be comparable to reported OS outcomes with ipilimumab in previously treated patients (median OS, 10.4 vs 10.7 months; 3-year OS, 21% vs 20%) [12]. Taken together, these results suggest that clinically relevant responses can be achieved with immunotherapy following progression on targeted therapy. In addition, observation of longer ppOS among patients who received initial ppRx with targeted therapy (74% single-agent BRAFi) compared with initial ppRx with treatments other than immunotherapy or targeted therapy is consistent with the findings of recent studies suggesting that reinitiation of BRAFi and/or MEKi following PD can provide clinical benefit [29].

These findings introduce an additional layer of complexity in the interpretation of treatment effects related to OS outcomes reported in randomized controlled trials. It should be noted that a significant proportion of patients across treatment cohorts received multiple lines of ppRx. Among patients who received ppRx with immunotherapy, more than 90% received ipilimumab as their initial ppRx, whereas anti–PD-1 therapy was predominantly used after failure of initial ppRx. Results from a recent study, albeit in the treatment-naive setting, suggest more favorable outcomes with anti–PD-1 followed by ipilimumab compared with the reverse sequence [30]. Proper interpretation of OS outcomes will require consideration of patient and disease characteristics as well as the distribution and sequence of treatments received following progression.

Strengths of this pooled analysis include the large number of patients from studies conducted in similar populations, sufficient follow-up for robust survival analyses, and prospective data collection using standardized schedules and methods. Further, the current analysis is largely based on data from randomized studies, wherein patients with similar baseline prognostic factors received a reference first-line and experimental treatment; this analysis is, therefore, less subject to the bias inherent in retrospective analyses of postprogression survival using data from patients treated in routine clinical practice where treatment decisions were based on physician and individual patient preferences [19–22]. However, as a retrospective exploratory analysis our analysis is also subject to limitations. The pooled dataset included patients enrolled in nonrandomized studies and original treatment assignment may be subject to selection bias. Also, choice of ppRx was not randomized or dictated by study protocols and may have been influenced in a nonuniform manner by changes in access to new treatments across the approximate 8-year time period over which the included studies were conducted.

## Conclusions

LDH, extent of disease, and ECOG PS remain significant prognostic factors following progression after treatment with cobimetinib plus vemurafenib, vemurafenib monotherapy, or dacarbazine in patients with *BRAF*^V600^-mutated metastatic melanoma. ppRx was also identified as an additional independent prognostic factor for OS following progression. Patients who progress following treatment with BRAFi and/or MEKi derive an OS benefit from subsequent treatment with immunotherapy or additional targeted therapy.

## Supplementary information

**Additional file 1: Table S1.** Demographic and clinical characteristics for patients included in the updated analysis of ppOS (N = 1027). **Table S2**. Initial postprogression treatment in all pooled patients. **Table S3.** Distribution of subsequent postprogression treatments according to initial postprogression treatment.

## Data Availability

Qualified researchers requesting access to de-identified patient-level data through the Clinical Study Data Request platform will be provided with accompanying clinical study documentation (protocol and any associated amendments, annotated case report form, reporting and analysis plan, dataset specifications, clinical study report). Researchers requesting access to clinical study documentation only, can do so via the following link: http://www.roche.com/research_and_development/who_we_are_how_we_work/clinical_trials/our_commitment_to_data_sharing/clinical_study_documents_request_form.htm

## Abbreviations

BRAFi: BRAF inhibitor
CI: Confidence interval
CNS: Central nervous system
CTLA-4: Cytotoxic T-lymphocyte-associated protein 4
ECOG PS: Eastern Cooperative Oncology Group performance status
IQR: Interquartile range
LDH: Lactate dehydrogenase
MEKi: MEK inhibitor
NE: Not estimable
OS: Overall survival
PD: Progressive disease
PD-1: Programmed death 1
ppOS: Postprogression overall survival
ppRx: Postprogression treatment
RPA: Recursive partitioning analysis
SLD: Sum of longest diameters
ULN: Upper limit of normal
