# Supplementary material for: Impact of initial treatment and prognostic factors on postprogression survival in BRAF-mutated metastatic melanoma treated with dacarbazine or vemurafenib ± cobimetinib: a pooled analysis of four clinical trials
Source: J Transl Med. 2020 Aug 3;18:294. doi: 10.1186/s12967-020-02458-x (PMC7397682; doi:10.1186/s12967-020-02458-x)
Supplement: Supplementary file 1 — Additional file 1: Table S1. Demographic and clinical characteristics for patients included in the updated analysis of ppOS (N = 1027). Table S2. Initial postprogression treatment in all pooled patients. Table S3. Distribution of subsequent postprogression treatments according to initial postprogression treatment. [file 12967_2020_2458_MOESM1_ESM.docx]

**Additional file 1**

**Table S1.** Demographic and clinical characteristics for patients included in the updated analysis of ppOS (N=1027)

| **Characteristic** | **All pooled patients**  **N=1027** | **Cobimetinib + vemurafenib**  **n=200** | **Vemurafenib monotherapy**  **n=562** | **Dacarbazine**  **n=265** |
| --- | --- | --- | --- | --- |
| Age, n (%) | (n=1027) | (n=200) | (n=562) | (n=265) |
| <65 y | 779 (75.9) | 156 (78.0) | 412 (73.3) | 211 (79.6) |
| ≥65 y | 248 (24.1) | 44 (22.0) | 150 (26.7) | 54 (20.4) |
| Sex, n (%) | (n=1027) | (n=200) | (n=562) | (n=265) |
| Male | 614 (59.8) | 126 (63.0) | 340 (60.5) | 148 (55.8) |
| Female | 413 (40.2) | 74 (37.0) | 222 (39.5) | 117 (44.2) |
| Race, n (%) | (n=1027) | (n=200) | (n=562) | (n=265) |
| White | 992 (96.6) | 181 (90.5) | 546 (97.2) | 265 (100.0) |
| Nonwhite | 35 (3.4) | 19 (9.5) | 16 (2.8) | 0 |
| Region, n (%) | (n=1027) | (n=200) | (n=562) | (n=265) |
| North America | 271 (26.4) | 48 (24.0) | 163 (29.0) | 60 (22.6) |
| Europe | 608 (59.2) | 120 (60.0) | 320 (56.9) | 168 (63.4) |
| Australia/New Zealand/Others | 148 (14.4) | 32 (16.0) | 79 (14.1) | 37 (14.0) |
| Baseline disease stage, n (%) | (n=1026) | (n=200) | (n=561) | (n=265) |
| Unresectable IIIC, M1a, or M1b | 343 (33.4) | 63 (31.5) | 189 (33.7) | 91 (34.3) |
| M1c | 683 (66.6) | 137 (68.5) | 372 (66.3) | 174 (65.7) |
| Baseline ECOG PS, n (%) | (n=1024) | (n=198) | (n=561) | (n=265) |
| 0 | 670 (65.4) | 142 (71.7) | 349 (62.2) | 179 (67.5) |
| 1 | 354 (34.6) | 56 (28.3) | 212 (37.8) | 86 (32.5) |
| Baseline LDH, n (%) | (n=963) | (n=188) | (n=529) | (n=246) |
| Normal | 510 (53.0) | 92 (48.9) | 281 (53.1) | 137 (55.7) |
| Elevated ≤2 × ULN | 293 (30.4) | 63 (33.5) | 155 (29.3) | 75 (30.5) |
| Elevated >2 × ULN | 160 (16.6) | 33 (17.6) | 93 (17.6) | 34 (13.8) |
| Baseline liver metastasis, n (%) | (n=1024) | (n=200) | (n=559) | (n=265) |
| Yes | 363 (35.4) | 75 (37.5) | 199 (35.6) | 89 (33.6) |
| No | 661 (64.6) | 125 (62.5) | 360 (64.4) | 176 (66.4) |
| ECOG PS at PD, n (%) | (n=948) | (n=181) | (n=526) | (n=241) |
| 0 | 505 (53.3) | 119 (65.7) | 271 (51.5) | 115 (47.7) |
| 1 | 360 (38.0) | 52 (28.7) | 211 (40.1) | 97 (40.2) |
| 2 | 56 (5.9) | 5 (2.8) | 29 (5.5) | 22 (9.1) |
| 3 | 23 (2.4) | 5 (2.8) | 13 (2.5) | 5 (2.1) |
| 4 | 4 (0.4) | 0 | 2 (0.4) | 2 (0.8) |
| Postprogression treatment, n (%) | (n=1027) | (n=200) | (n=562) | (n=265) |
| Immunotherapy | 218 (21.2) | 51 (25.5) | 116 (20.6) | 51 (19.2) |
| Targeted therapy | 82 (8.0) | 19 (9.5) | 35 (6.2) | 28 (10.6) |
| Other | 727 (70.8) | 130 (65.0) | 411 (73.1) | 186 (70.2) |
| Baseline SLD, mm | (n=1018) | (n=200) | (n=553) | (n=265) |
| Mean (SD) | 86.5 (74.6) | 84.9 (62.1) | 90.6 (85.2) | 79.3 (57.2) |
| Median (range) | 69.0 (9–1310) | 70.5 (10–398) | 70.0 (9–1310) | 66.0 (9–295) |

ECOG PS, Eastern Cooperative Oncology Group performance status; LDH, lactate dehydrogenase; PD, progressive disease; ppOS, postprogression overall survival; RPA, recursive partitioning analysis; SLD, sum of longest diameter; ULN, upper limit of normal.

**Table S2.** Initial postprogression treatment in all pooled patients

| **Treatment, n (%)** | **All pooled patients**  **N=1027** |
| --- | --- |
| Immunotherapy | 218 (21.2) |
| Ipilimumab | 200 (19.5) |
| Pembrolizumab | 8 (0.8) |
| Anti–PD-1 therapy NOS | 5 (0.5) |
| Anti–CTLA-4 therapy NOS | 2 (0.2) |
| Nivolumab | 2 (0.2) |
| Ganitumab | 1 (0.1) |
| Targeted therapy | 82 (8.0) |
| Dabrafenib | 28 (2.7) |
| Vemurafenib | 24 (2.3) |
| Trametinib | 9 (0.9) |
| BRAF inhibitor NOS | 8 (0.8) |
| Dabrafenib + trametinib | 6 (0.6) |
| Cobimetinib | 2 (0.2) |
| MEK inhibitor NOS | 2 (0.2) |
| MEK162 | 1 (0.1) |
| Pimasertib | 1 (0.1) |
| RO 5212054 | 1 (0.1) |
| Other | 727 (70.8) |
| Chemotherapy | 220 (21.4) |
| No treatment | 507 (49.4) |

CTLA-4, cytotoxic T lymphocyte–associated antigen 4; NOS, not otherwise specified; PD-1, programmed death receptor 1.

**Table S3.** Distribution of subsequent postprogression treatments according to initial postprogression treatment

|  | **Immunotherapy**  **n=218** | **Targeted therapy**  **n=82** | **Chemotherapy**  **n=220** |
| --- | --- | --- | --- |
| Anti–PD-1 therapy | 44 (20.2) | 12 (14.6) | 12 (5.5) |
| Chemotherapy | 28 (12.8) | 15 (18.3) | 43 (19.5) |
| BRAF inhibitor | 28 (12.8) | 12 (14.6) | 13 (5.9) |
| Anti–CTLA-4 therapy | 21 (9.6) | 24 (29.3) | 40 (18.2) |
| MEK inhibitor | 7 (3.2) | 10 (12.2) | 3 (1.4) |
| BRAF inhibitor + MEK inhibitor | 5 (2.3) | 0 | 1 (0.5) |
| Anti–PD-1 + anti–CTLA-4 therapy | 0 | 1 (1.2) | 0 |

CTLA-4, cytotoxic T lymphocyte–associated antigen 4; PD-1–programmed death receptor 1.
